# Supplementary material for: Extinction cascades, community collapse, and recovery across a Mesozoic hyperthermal event
Source: Nat Commun. 2024 Oct 4;15:8599. doi: 10.1038/s41467-024-53000-2 (PMC11452722; doi:10.1038/s41467-024-53000-2)
Supplement: Supplementary file 1 — Supplementary Information [file 41467_2024_53000_MOESM1_ESM.pdf]

Supplementary information for Extinction cascades, community collapse, and recovery across a Mesozoic hyperthermal event

Dunhill *et al.*

Table S1. Trophic guilds with associated taxonomic and ecological traits

Guild	Clade	Motility	Tiering	Feeding	Size	Calcification
Ammonite_1	ammonite	slow_swimmer	pelagic	predator	small	moderate
Ammonite_2	ammonite	slow_swimmer	pelagic	predator	medium	moderate
Ammonite_3	ammonite	slow_swimmer	pelagic	predator	large	moderate
Belemnite_1	belemnite	fast_swimmer	pelagic	predator	large	moderate
Belemnite_2	belemnite	fast_swimmer	pelagic	predator	very_large	moderate
Belemnite_3	belemnite	fast_swimmer	pelagic	predator	gigantic	moderate
Bivalve_1	bivalve	slow_crawler	shallow_infaunal	suspension	small	moderate
Bivalve_2	bivalve	facultative_unattached	surficial	deposit	medium	moderate
Bivalve_3	bivalve	facultative_unattached	shallow_infaunal	mining	small	moderate
Bivalve_4	bivalve	slow_crawler	shallow_infaunal	mining	medium	moderate
Bivalve_5	bivalve	slow_crawler	shallow_infaunal	mining	small	moderate
Bivalve_6	bivalve	slow_crawler	shallow_infaunal	mining	tiny	moderate
Bivalve_7	bivalve	facultative_attached	deep_infaunal	suspension	medium	moderate
Bivalve_8	bivalve	facultative_unattached	deep_infaunal	suspension	medium	moderate
Bivalve_9	bivalve	slow_crawler	deep_infaunal	suspension	large	moderate
Bivalve_10	bivalve	slow_crawler	deep_infaunal	suspension	medium	moderate
Bivalve_11	bivalve	nonmotile_attached	erect	suspension	very_large	moderate
Bivalve_12	bivalve	nonmotile_attached	semi-infaunal	suspension	large	moderate
Bivalve_13	bivalve	facultative_unattached	shallow_infaunal	suspension	medium	moderate
Bivalve_14	bivalve	facultative_unattached	shallow_infaunal	suspension	small	moderate
Bivalve_15	bivalve	facultative_attached	surficial	suspension	large	moderate
Bivalve_16	bivalve	facultative_attached	surficial	suspension	medium	moderate
Bivalve_17	bivalve	facultative_attached	surficial	suspension	small	moderate
Bivalve_18	bivalve	facultative_attached	surficial	suspension	tiny	moderate
Bivalve_19	bivalve	facultative_unattached	surficial	suspension	large	moderate
Bivalve_20	bivalve	nonmotile_attached	surficial	suspension	medium	moderate
Bivalve_21	bivalve	nonmotile_attached	surficial	suspension	small	moderate
Bivalve_22	bivalve	nonmotile_unattached	surficial	suspension	large	moderate
Bivalve_23	bivalve	nonmotile_unattached	surficial	suspension	small	moderate
Bivalve_24	bivalve	facultative_unattached	surficial	suspension	small	moderate
Brachiopod_1	brachiopod	nonmotile_attached	surficial	suspension	small	heavy
Brachiopod_2	brachiopod	facultative_unattached	shallow_infaunal	suspension	small	light
Bryozoan_1	bryozoan	nonmotile_attached	surficial	suspension	tiny	heavy
Crinoid_1	crinoid	facultative_attached	erect	suspension	medium	heavy
Crinoid_2	crinoid	facultative_attached	erect	suspension	small	heavy
Crustacean_1	crustacean	fast_crawler	semi-infaunal	predator	large	moderate
Crustacean_2	crustacean	fast_crawler	semi-infaunal	predator	medium	moderate
Crustacean_3	crustacean	fast_crawler	semi-infaunal	predator	medium	moderate
Crustacean_4	crustacean	fast_crawler	semi-infaunal	predator	small	moderate
Crustacean_5	crustacean	fast_crawler	surficial	predator	large	moderate
Crustacean_6	crustacean	fast_crawler	surficial	predator	small	moderate
Crustacean_7	crustacean	fast_crawler	surficial	predator	medium	moderate
Echinoid_1	echinoid	Slow_crawler	surficial	predator	large	moderate
Fish_1	fish	fast_swimmer	pelagic	predator	large	light
Gastropod_1	gastropod	slow_crawler	surficial	grazer	tiny	moderate

Gastropod_2	gastropod	slow_crawler	surficial	predator	tiny	moderate
Gastropod_3	gastropod	facultative_unattached	surficial	suspension	tiny	moderate
Gastropod_4	gastropod	nonmotile_unattached	surficial	suspension	tiny	moderate
Ophiuroid_1	ophiuroid	slow_crawler	surficial	predator	medium	heavy
Scaphopod_1	scaphopod	facultative_unattached	semi-infaunal	predator	small	moderate
Serpulid_1	serpulid	nonmotile_attached	surficial	suspension	small	moderate
Soft_1	soft_indet	slow_crawler	surficial	grazer	medium	light
Soft_2	soft_indet	slow_crawler	surficial	grazer	small	light
Soft_3	soft_indet	slow_crawler	shallow_infaunal	mining	small	light
Soft_4	soft_indet	slow_crawler	shallow_infaunal	suspension	small	light
Soft_5	soft_indet	slow_crawler	deep_infaunal	suspension	small	light
Basal_node	primary	primary	primary	primary	primary	primary

Table S2. Structural network metrics and network motifs for the 4 time bins spanning the ETE and recovery interval.

Metric	Pre-extinction	Post-extinction	Early recovery	Late recovery
Size/richness	48	21	29	45
Connectance	0.13	0.19	0.15	0.14
Max Trophic Level	3.3	3.5	3.5	3.7
Generality	0.2	0.24	0.2	0.19
Vulnerability	0.11	0.14	0.14	0.11
S1: Number of linear chains	0.33	0.27	0.21	0.25
S2: Omnivory	0.33	0.27	0.21	0.25
S4: Apparent competition	0.9	0.38	0.42	0.7
S5: Direct competition	0.32	0.18	0.25	0.33

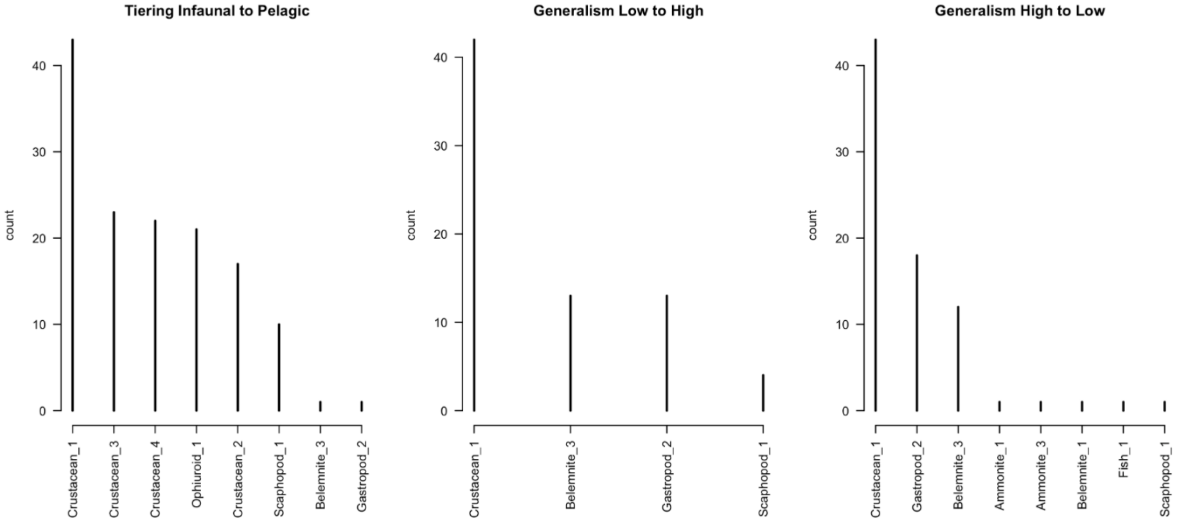

Figure S1. Secondary extinction frequency of guilds in the 3 best supported models for secondary extinction cascades.
